# Moral Bargain Hunters Purchase Moral Righteousness When it is Cheap: Within-Individual Effect of Stake Size in Economic Games

**DOI:** 10.1038/srep27824

**Published:** 2016-06-14

**Authors:** Toshio Yamagishi, Yang Li, Yoshie Matsumoto, Toko Kiyonari

**Affiliations:** 1 Brain Science Institute, Tamagawa University, Machida 194-8610, Japan; 2 Graduate School of International Corporate Strategy, Hitotsubashi University, Tokyo 101-8439, Japan; 3 School of Social Informatics, Aoyama Gakuin University, Sagamihara 252-5258, Japan

## Abstract

Despite the repeatedly raised criticism that findings in economic games are specific to situations involving trivial incentives, most studies that have examined the stake-size effect have failed to find a strong effect. Using three prisoner’s dilemma experiments, involving 479 non-student residents of suburban Tokyo and 162 students, we show here that stake size strongly affects a player’s cooperation choices in prisoner’s dilemma games when stake size is manipulated within each individual such that each player faces different stake sizes. Participants cooperated at a higher rate when stakes were lower than when they were higher, regardless of the absolute stake size. These findings suggest that participants were ‘moral bargain hunters’ who purchased moral righteousness at a low price when they were provided with a ‘price list’ of prosocial behaviours. In addition, the moral bargain hunters who cooperated at a lower stake but not at a higher stake did not cooperate in a single-stake one-shot game.

The use of economic game experiments has dramatically increased in the past few decades in a variety of disciplines of social and biological sciences including psychology^1^,^2^, economics^3^,^4^,^5^, political science^6^, sociology^7^, anthropology^8^, evolutionary biology^9^,^10^, and neuroscience^11^,^12^. Such games are useful tools with which to examine human psychological dispositions, such as social preferences, cognitive mechanisms, and beliefs about the behavioural principles of their interaction partners^12^,^13^,^14^,^15^,^16^,^17^,^18^,^19^,^20^,^21^. However, the issue of the generalisability of the findings has not been fully resolved^22^,^23^. Particularly, one repeatedly raised criticism of economic game experiments is that the psychological and cognitive influences found in economic games are limited to situations involving monetarily trivial incentives, most often with a value of a few hours’ wages at most^24^,^25^,^26^,^27^,^28^,^29^,^30^,^31^. The psychological factors are suspected not to exert a strong effect on game players when the monetary incentives reach a substantial level. Experimental game researchers have since responded to these criticisms by conducting various experimental games that manipulated monetary stake size. With notable exceptions^25^,^32^,^33^, the results of these studies generally concur with the conclusion that stake size does not strongly influence participants’ game behaviour, at least until it is raised to the value of a few weeks’ wages^32^,^33^,^34^,^35^,^36^,^37^,^38^,^39^,^40^,^41^. Camerer and Hogarth^42^ reviewed a wide range of game and non-game experiments that involved different incentives and concluded that incentive size usually has little effect on mean game behaviour, whereas larger stakes tend to reduce the variance among individual players. Some of the studies were conducted in low-income countries to increase the upper limit of the stake to the level of a few weeks’ or even a few months’ earnings. For example, an ultimatum game (UG) experiment conducted in the Slovak Republic^41^ raised the upper limit of the stake to 1,500 Slovak crowns (compared with an average monthly income of 5,500), and yet the average offer was hardly affected as stake size increased, while the proportion of rejection of unfair offers significantly declined. Another UG experiment in Indonesia^32^, in which the highest stake size was equivalent to three months’ income, found a similar pattern whereby the proposal was hardly affected by stake size and the proportion of rejections declined. This was also the case in studies conducted in France and Romania (where a high stake was worth a 20-month student scholarship)^33^. Another study^43^ conducted in an Indian village varied stake size from 20 to 20,000 rupees (1.6–1,600 wage hours) and found both a steady decline in offers and in the rejection rate. In a trust game experiment conducted in Bangladesh^25^, in which the researchers manipulated stake size from 40 Taka to 1,000 Taka, a sum approximating 4.8% (18 days) of per capita gross national income, trust choices significantly declined as stake size increased, whereas the return did not.

Recent studies that have tested the effect of stakes have predominantly used the UG, and less often, the dictator game and the trust/investment game. Despite the early interest in the stake-size effect in prisoner’s dilemma games (PDGs), few studies of stake size in PDGs have been carried out recently. A study of the sequential PDG^44^ found no significant effect of an increase in stake size in the first player’s cooperation rate, whereas the second player’s responses to cooperation by the first player declined in the larger-stake game. In another study of a three-person public-goods game with and without punishment, stake size had little effect on either cooperation or punishment^45^. Even when stakes ranged from a value of US$200–16,000 in the TV show ‘Friend or Foe’, in which a variant of the one-shot PDG was played, the cooperation rate did not vary across large differences in stake size^46^. An analysis of a similar British TV program, ‘Golden Balls’, in which stake size varied from an equivalent of US$20,000–US$175,000, the cooperation rate declined throughout the lower half of the stake distribution and stabilised in the upper half^47^. The findings of these studies generally indicate that stake size does not strongly affect game players’ behaviour up to a value of a few weeks’ earnings. Another related literature concerns the size of extrinsic incentives including both rewards and punishments^48^,^49^,^50^ which indicates that weak extrinsic monetary incentives reduce voluntary cooperation because explicit extrinsic incentives transform peoples’ perception of the task from that of moral obligation to contract enforcement. In general, explicit sanctions may ruin the intrinsic motivation for cooperation (over-justification effect)^51^. Although this literature raises interesting theoretical issues concerning the effect of extrinsic incentives, it does not directly predict the effect of stake size in voluntary social exchanges that involve no extrinsic incentives.

One notable feature common to the aforementioned studies of stake size is that all but one UG study^33^ compared participants’ behaviour using a between-individual design. That is, each participant experienced one stake size, which differed between participants. The only exception is a within-individual comparison of stake size that found a substantial decline in the minimum acceptance level in the UG as stake size increased to a level equivalent to a 20-month student scholarship^33^. No studies examined the stake-size effect when each participant faces different stake sizes within the range that is typically used in economic game experiments. To the best of our knowledge, ours is the first PDG study that compares the cooperation rate by using a within-participant manipulation of stake size.

Between-individual and within-individual manipulations of stake size differ in that the latter provides a way for the player to combine low-stake choices and high-stake choices to form a portfolio for maximizing total utility. For example, players may use the low-stake games to explore if a mutually rewarding relationship can be initiated by showing initiative when the cost is low, expecting to derive larger benefits in high-stake games. Furthermore, players may expect that their partners will be more willing to cooperate when the stake is small; therefore, it is safe to initiate cooperation when the stake is small. Such expectations of greater cooperation of partners in small-stake games will further enhance a player’s willingness to cooperate when the stake is small. This process of testing the waters in small-stake games as a strategy to establish a mutually rewarding relationship in large-stake games will generate a within-individual stake-size effect.

Alternatively, players may try to maximise utilities derived from engaging in morally justified behaviour and from material gains. One possible reason for the weak stake-size effect, when it is manipulated between participants, is that the utility of morally preferred behaviour increases with monetary gains (i.e. low guilt versus low monetary gain compared to high guilt versus high monetary gain). In contrast, when each participant faces several games of different stake sizes, some game players may act as ‘moral bargain hunters’ who ‘purchase’ moral righteousness when the cost of such action is low. Therefore, within-individual manipulation would produce a stronger stake-size effect. This moral bargain hunter hypothesis is based on the assumption that some people prefer perceiving themselves as morally righteous person; if one does not have this preference, one will not seek a moral bargain. The moral bargain hunter hypothesis also assumes that acting in a prosocial manner provides ‘evidence’ for perceiving oneself as morally righteous.

To test if stake size has a substantial effect when each individual makes decisions in a PDG with varying stake sizes, we conducted two PDG experiments (Studies 1 and 2) with differing stake sizes and a single-stake PDG (Study 3), which provided additional support for this hypothesis. We found for the first time that a strong effect exists when stake size is manipulated within individuals. Furthermore, the within-individual stake size effect exists even when the risk of exploitation by an uncooperative partner is completely eliminated. This finding ruled out testing-the-waters-when-risk-is-small explanation as the psychological mechanism of the observed within-individual stake-size effect.

## Study 1: Repeated one-shot game with multiple roles

Each participant played an exchange format PDG nine times, in which they decided whether they would provide an endowment (JPY 300, 800, 1,500) to a randomly assigned partner. The player acted in each of three roles—the sequential-first player role, the sequential-second player role, and the simultaneous role. Each of the nine games represented a unique combination of the protocol and the stake. The provided endowment was doubled and then awarded to the partner.

In the simultaneous protocol, two players made decisions without knowing if their respective partners would cooperate. In the sequential protocol, one of the two players—the first player—made a decision without knowing what the other player—the second player—would do; subsequently, the second player made a decision knowing if the first-player cooperated or not. The risk of exploitation existed if the player cooperated in the simultaneous protocol or the sequential protocol as a first player. On the other hand, when the player made a decision in the sequential protocol as the second player, there was no risk of exploitation because the partner’s decision was known.

**Figure 1:**
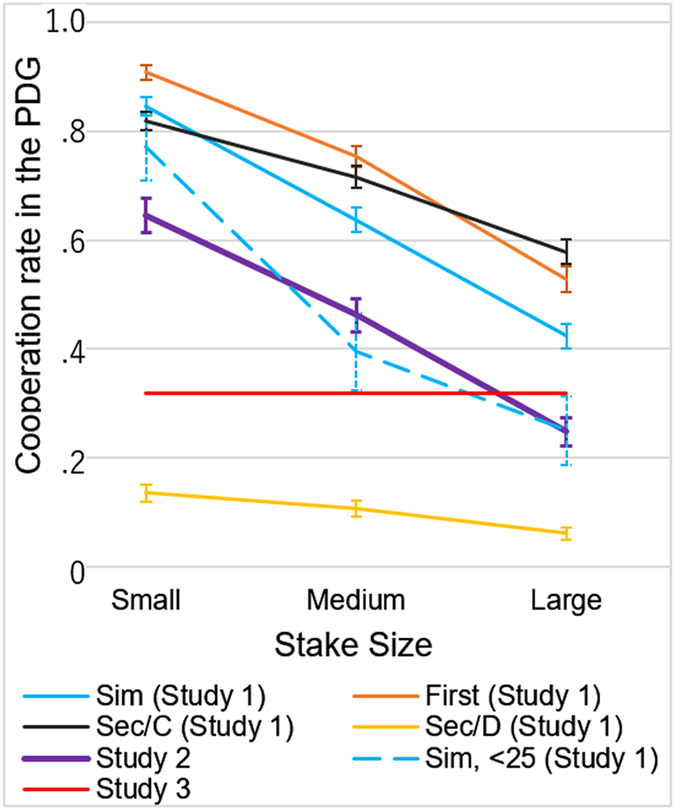
Cooperation rates in Study 1 in each role and Study 2 as a function of stake size, and cooperation rate in Study 3. The purple line is for the cooperation rate in Study 2. The red line represents the cooperation rate in Study 3, to be compared with the cooperation rates in the other games. The other lines are for Study 1. The blue line (Sim) is for the cooperation rate in the simultaneous role and the dotted blue (Sim, <25) line is for the same cooperation rate among participants aged under 25. The brown line (First) is for the first player in the sequential game, the yellow line (Sec/D) is for the second player who faced the first player’s defection, and the black line (Sec/C) is for the second player who face the first player’s cooperation. Error bars represent 95% confidence intervals.

**Figure 2:**
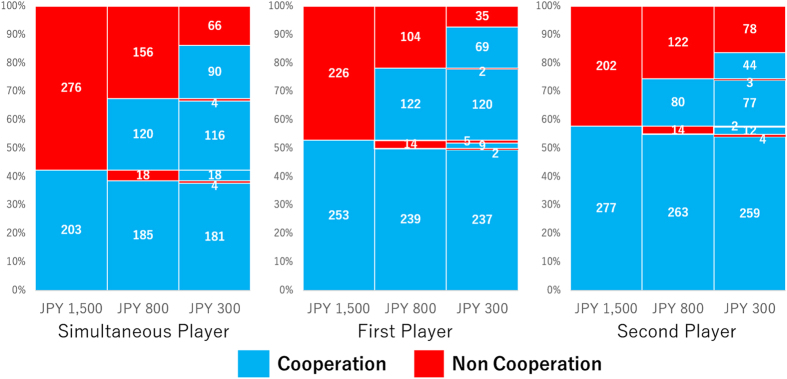
Frequencies of cooperators and non-cooperators in each stake condition and player role. The second column represents the frequencies of cooperators and non-cooperators for each action in the first column, and the third column represents the frequencies of cooperators and non-cooperators for each action in the second column.

Figure 1 shows the cooperation rate at the three levels of stake size in each role. A far stronger stake-size effect than that typically observed in experiments using between-individual manipulation was observed for all roles, except the second player’s response to the first player’s defection, for which the overwhelming majority of the players defected. Stake size’s effect on cooperation rate in each role was highly significant in the first three roles (generalized linear mixed model analysis: simultaneous role, *F*_(2, 1434)_ = 82.90, *P* < 0.0001; first player role, *F*_(2, 1434)_ = 76.88, *P* < 0.0001; second player role in which the first player cooperated, *F*_(2, 1434)_ = 32.04, *P* < 0.0001). The cooperation rates were extremely high in all three conditions when the stake size was small (84.6% in the simultaneous condition, 90.8% in the first player condition, and 81.8% in the second/C condition). Even the overwhelming majority of the participants who did not cooperate when the stake was large (JPY 1,500) cooperated when the stake was JPY 300 (74.6% in the simultaneous role, 83.6% in the first player’s role, and 59.9% in the second player’s role facing the cooperative first player) (Fig. 2).

The results of Study 1 unequivocally show that stake size has a strong effect on cooperation in a PDG within a range of stake sizes (approximately $2.50– $12.50) with which the stake size effect has rarely been observed in previous studies. The stake-size effect existed even when the participants played the role of the second player and faced a cooperative first player, that is, when the risk of being exploited by an uncooperative partner was absent. However, some features of the design of Study 1 prevent us from generalising the findings. Specifically, the order of the within-participant manipulation of the game conditions and the stake-size conditions were fixed (see the Method section). Furthermore, while the participant played the game three times with each stake size, they played these three trials in different roles – as a simultaneous player, a first player in a sequential game, and a second player in a sequential game. Although Fig. 1 shows that the stake-size effect existed in each role, we cannot eliminate the possibility that the experience of playing these different roles somehow affected the participant’s responses to different stake sizes. To address these limitations, we decided to re-run the study with a modified design (Study 2). In addition, we substantially reduced the overall stake size to examine whether the within-participant stake-size effect exists at even lower stakes.

### Study 2: Replication of the within-individual effect with a modified design

Study 2 adopted the same basic design as Study 1, except that only the simultaneous role was used. Each participant played a binary PDG 30 times, 10 times for each endowment of JPY 100, 200, or 400, each time with a different partner. The order of the endowment size was randomised. We succeeded in replicating the strong within-participant stake-size effect of Study 1 (purple line in Fig. 1). While the overall levels of cooperation were lower than those observed in the comparable condition of the simultaneous game in Study 1 (blue line in Fig. 1), the stake size had a strong effect (generalized linear mixed model analysis: *F*_(2,322)_ = 242.92, *P* < 0.0001). Because the prosociality is known to increase with age^1^, and thus the low cooperation rate in Study 2 could have resulted from the younger sample, we compared the cooperation rates in Study 2 with those of a similar age group in Study 1 (ages 20–24 years). The cooperation rates for this age group in Study 1 (broken blue line in Fig. 1) were similar to those in Study 2. The overall cooperation rate of the young participants in Study 1 (N =  48, M = 0.47, SD = 0.35) was not significantly different from that in Study 2 (M = 0.45, SD = 0.33; *t*_(208)_ = 0.11, *P* = 0.866). We thus suggest that the lower cooperation rates in Study 2 were due to age differences in the samples and not to the smaller stake size.

The results of Studies 1 and 2 thus indicate that the relative but not absolute stake size affects participants’ cooperation rates. Study 3 examined if the within-individual stake-size effect is generated by an increase in cooperation when the stakes are relatively low, not by a decrease in cooperation when the stakes are relatively high, compared to the default cooperation level in a one-shot single-stake game. Given the extremely high cooperation rates (above 80%) observed in the small-stake condition of Study 1, it is more likely that relatively low stakes enhanced cooperation rather than relatively high stakes reduced cooperation. However, this higher-than-usual cooperation rate could have resulted from using an older and generally more cooperative sample in which the majority of participants were older than students commonly used in other studies. To eliminate this possibility, we compared the results of Study 1 with those of another study that used the same sample, that is, Study 3. Study 3 was a one-shot single-stake game in which all participants faced a PDG with a stake size of JPY 1,000. Study 3 adopted a continuous-choice design, according to which each participant decided on the level of cooperation up to a stake of JPY 1,000, in increments of 100.

### Study 3. One-shot single-stake PDG

Both the moral bargain hunter hypothesis and the testing the waters hypothesis predict that the cooperation rate will be higher when the stake size is small compared to the benchmark cooperation rate in the single-stake game. The result of Study 3 with a single-stake of JPY 1,000 confirmed that the within-participant stake-size effect was caused by an increase in cooperation as the stake decreased, not caused by a decrease in cooperation as the stake increased (from JPY 1,000 to JPY 1,500). The cooperation rate in the single-stake game in Study 3 was 0.319 (±0.030 95% confidence interval). The cooperation rates in the low-stake and medium-stake simultaneous games were much higher than this benchmark cooperation rate, whereas the cooperation rate in the high-stake simultaneous game was relatively close to the benchmark cooperation rate in Study 3 (Fig. 1). Participants who fully cooperated in Study 3 (n = 40) also cooperated at high rates in Study 1 regardless of the stake size (90.0% in low-stake, 90.0% in medium-stake, and 82.5% in high-stake games), whereas those who did not cooperate at all in Study 3 (n = 151) greatly changed their choices depending on the stake size (71.5% in low-stake, 49.0% in medium-stake, and 24.5% in high-stake games). Furthermore, the cooperation level in Study 3 correlated more strongly with the cooperation levels in the high-stake condition (*r* = 0.486, *P* < 0.0001) and the medium-stake condition (*r* = 0.406, *P* < 0.0001) of Study 1 than with the cooperation level in the low-stake condition (*r* = 0.294, *P* < 0.0001). When the one-shot cooperation level was predicted in a regression analysis by the cooperation levels at three levels of stake size, only the effect of the high-stake cooperation remained highly significant (*β* = 0.316, *t* = 6.87, *P* < 0.0001). Neither the effect of the medium-stake cooperation (*β* = 0.105, *t* = 1.77, *P* = 0.077) nor the effect of the low-stake cooperation (*β* = 0.036, *t* = 0.54, *P* = 0.588) was significant. This result suggests that our participants made decisions in the high-stake condition using the same psychological mechanism they used in the one-shot game, whereas the psychological mechanism they used in the low-stake condition differed substantially from what they used in the one-shot game.

## Discussion

For the first time, we found a strong stake-size effect in two experiments in which stakes were compared within individuals. We proposed two psychological mechanisms to predict this within-individual stake-size effect. The presence of the stake-size effect in the second/C condition eliminates the testing-the-waters hypothesis. Only the moral bargain hunter hypothesis remains according to which some of the participants cooperate when the cost of cooperation is relatively low. We also found that the decisions participants made when the stake was relatively high was consistent with the decisions they made in the one-shot game; however, their one-shot decisions were not so much consistent with their decisions in the low-stake game. Although caution is advisable when comparing the cooperation levels in Study 1 and Study 3 because of the difference in the decision scheme—binary choice in Study 1 and continuous choice in Study 3—this difference should not falsify our conclusion that our participants played the high-stake game in Study 1 in the same way that they played the no-stake-comparison game in Study 3, whereas they played the low-stake game in a way not consistent with the no-stake comparison game.

The moral bargain hunter hypothesis of the within-individual stake-size effect attributes the weak or no stake-size effect in previous studies to the absence of a bargain (i.e., the opportunity to purchase moral righteousness at a low cost). In a single-stake game, moral righteousness is sold at a fixed price. The utility of being morally righteous and pecuniary cost are thus difficult to compare in a single-stake game in which players cannot hunt for a bargain. The within-individual comparison of stake sizes provided prices of moral righteousness to compare, and many participants—even those who did not cooperate at all in a single-stake game—purchased moral righteousness when the monetary stake was relatively low. Exposure to different stake sizes in the current study design may have made the participant aware that his or her behaviour was the target of the study, and this design feature thus may have enhanced participants’ awareness of the utility of being or appearing prosocial. This heightened awareness of their behaviour being relevant to social evaluation might have triggered the process of moral bargain hunting. If this speculation is true, does it imply that the within-individual stake-size effect is an experimental artefact? The answer can be both yes and no, depending on whether people care about being morally righteous in real life. If they do care, the finding is not an artefact because the within-individual manipulation simply reproduces in the laboratory what exists in daily life. If most people do not care, our findings are an experimental artefact that does not have relevance to real-life situations. We believe that many people, although not all, do care about being morally righteous. Another related topic worthy of further research is cultural differences in the within-individual stake-size effect, which may reflect cultural differences in the rigor versus laxity of norm enforcement^52^ and the resulting demands for moral righteousness. One piece of missing information in the current study is the evidence that only those who care about their self or public image improved cooperative choices when stakes are low. Future studies should address this issue.

Finally, it should be emphasized that we are not concluding that humans are always trading pecuniary gain for moral righteousness. The following thought experiment inspired by Falk and Szech^53^ suggests that the majority of people may not exhibit a within-individual stake-size effect when the choice involves some non-pecuniary values. Participants in this thought experiment face a decision of paying an increasingly larger monetary cost for saving the life of a mouse, a dog, or a man, corresponding to the three levels of stake size used in this study. The pecuniary cost for saving a mouse, a dog, or a man has been pre-selected at the rates according to which between-participant comparison does not produce a differential probability for bearing the monetary cost. We speculate that few people will purchase moral righteousness by paying little money to save the life of a mouse while not paying a large amount of money to save the life of a man. This simple thought experiment suggests that our conclusion will not apply when something of supreme value, such as human life, is involved. The guilt of not saving a human life cannot be alleviated by saving the life of a mouse. Identifying the situations in which a within-individual stake-size effect occurs is an important topic for future research.

## Methods

### Approval and informed consent.

Studies 1 and 3 were conducted at the Brain Science Institute, Tamagawa University, and Study 2 was conducted at the Centre for Experimental Research in the Social Sciences, Hokkaido University. The study protocol was approved by the Ethics Committees of the respective institute where the studies were conducted, and the studies were carried out in accordance with the approved protocol which met the requirements of the Declaration of Helsinki. An informed consent form was signed by each participant.

### Study 1

Non-student residents (N = 479, 230 women and 249 men) of a suburban city of Tokyo and its surrounding localities participated in Study 1. Participants were evenly distributed according to age (20–59 years, *M* = 40.09, *SD* = 10.69, as of January of 2012) and gender. Four to 10 people participated in one session. Each participant played a binary PDG nine times, with a different partner each time. Participants were endowed with JPY 300, 800, or 1,500 (≈US$2.50, 6.70, or 12.50), which varied between trials, and decided whether they wished to provide that endowment to their partner or keep it for themselves. When the endowment was provided, the partner received double its value. For example, in a trial in which the size of the endowment was JPY 1,500, the partner received JPY 3,000 when the participant provided the endowment to the partner. Similarly, the participant received JPY 3,000 if the partner provided his/her endowment to the participant. When the participant instead kept the endowment, he/she earned the original endowment. Each participant played the game with the simultaneous protocol three times, as a first player in the sequential protocol three times, and as the second player three times. The strategy method was used when the participant played as a second player; that is, the participant decided whether to provide or keep his/her endowment twice in each trial, assuming the first player had once decided to provide the endowment and once to keep it. Participants first played the simultaneous game with stakes of JPY 300, 800, and 1,500 in that order, then played the sequential game in the first player’s role with the three stake sizes in the same order, and finally played the sequential game in the second player’s role using the strategy method with the same stake sizes in the same order. No feedback was provided when the participant played the role of a simultaneous player or a first player.

### Study 2

Japanese college students (N = 162, 66 women, mean age 19.8 years, aged 18–30 years, 95% younger than 25 years) participated in a PDG. Six to 14 people participated in one session in a laboratory equipped with 16 fully isolated booths. The instructions were provided as in Study 1. Only the simultaneous protocol was used. Each participant played a binary PDG 30 times, with a different partner each time. The participant was endowed with JPY 100, 200, or 400, which randomly varied between trials, and decided whether to give that endowment to their partner or keep it for themselves. As before, when the endowment was donated, the partner received twice its basic value. Each participant played the game 30 times, 10 times at each stake size. No feedback was provided after each trial.

### Study 3

We used the same pool of non-student residents as in Study 1. Although 479 people participated in Study 1, only 434 of them also participated in Study 3. The results reported here are based on the 434 participants who took part in both studies. Six to 10 people participated in one session. They played the following one-shot, simultaneous PDG in isolated booths. The participants received JPY 1,000 and decided how much of the money they wished to provide to their partner, in increments of JPY 100. The remaining endowment was theirs to keep. The partner received twice the amount of the money provided by the participant.

## Additional Information

**How to cite this article**: Yamagishi, T. *et al*. Moral Bargain Hunters Purchase Moral Righteousness When it is Cheap: Within-Individual Effect of Stake Size in Economic Games. *Sci. Rep.* **6**, 27824; doi: 10.1038/srep27824 (2016).

## Supplementary Material

Supplementary Information

Supplementary Information
